# RETRACTED: Ruan et al. Regulation of Long Non-Coding RNA-Dreh Involved in Proliferation and Migration of Hepatic Progenitor Cells during Liver Regeneration in Rats. *Int. J. Mol. Sci.* 2019, *20*, 2549

**DOI:** 10.3390/ijms26178479

**Published:** 2025-08-31

**Authors:** Zhiyan Ruan, Manxiang Lai, Ling Shang, Xiangliang Deng, Xinguo Su

**Affiliations:** 1 School of Pharmacy, Guangdong Food & Drug Vocational College, Guangzhou 510520, China; ruanzy@gdyzy.edu.cn (Z.R.); laimx@gdyzy.edu.cn (M.L.); shangl@gdyzy.edu.cn (L.S.); 2 School of Chinese Medicine, Guangdong Pharmaceutical University, Guangzhou 510006, China; dxl@gdpu.edu.cn

The journal retracts the article titled “Regulation of Long Non-Coding RNA-Dreh Involved in Proliferation and Migration of Hepatic Progenitor Cells during Liver Regeneration in Rats” [1], cited above.

Following publication, concerns were brought to the attention of the Editorial Office regarding image duplication within this article [1].

Adhering to our standard procedure, the Editorial Office and Editorial Board conducted an investigation that confirmed a duplication within the Western blot bands presented in Figures 3B and 6B. The authors fully cooperated with the investigation and requested that this publication be withdrawn. Consequently, the Editorial Board accepted the authors’ requests and decided to retract this publication [1], as per MDPI’s retraction policy (https://www.mdpi.com/ethics#_bookmark30 (accessed on 1 January 2020)).

This retraction was approved by the Editor-in-Chief of the *IJMS* journal. The authors agreed to this retraction.
